# Effect of physical activity and different exercise modalities on glycemic control in people with prediabetes: a systematic review and meta-analysis of randomized controlled trials

**DOI:** 10.3389/fendo.2023.1233312

**Published:** 2023-09-28

**Authors:** Miquel Bennasar-Veny, Narges Malih, Aina M. Galmes-Panades, Ivonne C. Hernandez-Bermudez, Natalia Garcia-Coll, Ignacio Ricci-Cabello, Aina M. Yañez

**Affiliations:** ^1^ Global Health and Lifestyles Research Group, Health Research Institute of the Balearic Islands (IdISBa), Palma, Spain; ^2^ CIBER de Epidemiología y Salud Pública (CIBERESP), Institute of Health Carlos III, Madrid, Spain; ^3^ Department of Nursing and Physiotherapy, University of the Balearic Islands (UIB), Palma, Spain; ^4^ Research Group on Global Health, University of the Balearic Islands (UIB), Palma, Spain; ^5^ CIBER of Physiopathology of Obesity and Nutrition (CIBEROBN), Instituto de Salud Carlos III, Madrid, Spain; ^6^ Physical Activity and Sport Sciences Research Group (GICAFE), Institute for Educational Research and Innovation (IRIE), University of the Balearic Islands, Palma, Spain; ^7^ Research Group in Primary Care and Promotion—Balearic Islands Community (GRAPP-caIB), Health Research Institute of the Balearic Islands (IdISBa), Palma, Spain; ^8^ Primary Care Research Unit of Mallorca (IB-Salut), Balearic Health Service, Palma de Mallorca, Spain; ^9^ Research Network on Chronicity, Primary Care, and Health Promotion (RICAPPS), Institute of Health Carlos III, Madrid, Spain

**Keywords:** prediabetes, exercise, aerobic training, resistance training, interval training

## Abstract

**Background:**

Numerous studies have shown the beneficial effects of exercise on glycemic control in people with prediabetes. However, the most effective exercise modality for improving glycemic control remains unclear. We aimed to assess which exercise training modality is most effective in improving glycemic control in a population with prediabetes.

**Methods:**

We conducted searches in Pubmed/MEDLINE, EMBASE, SPORTDiscus, Web of Science, PEDro, BVS, and the Cochrane Library from inception to June 2022. Included studies reported fasting plasma glucose (FPG), glycated hemoglobin (HbA1c), and 2-hour postprandial (2hPP) levels and implemented an exercise program lasting at least 12 weeks in adults with prediabetes. We performed a direct meta-analysis using a random-effects model and a network meta-analysis. Cochran’s Q statistic and the inconsistency I^2^ test were used to assess the heterogenicity between studies.

**Results:**

Twenty trials were included, with 15 trials (comprising 775 participants with prediabetes) combined in the meta-analysis, and 13 in the network meta-analysis. The meta-analysis results did not show a statistically significant reduction in fasting plasma glucose (FPG) after aerobic training (AT) intervention compared to a control group (mean (95%CI) difference = -5.18 (-13.48; 3.12) mg/dL, Z=1.22, p=0.22). However, a difference of -7.25 (-13.79; -0.71) mg/dL, p=0.03, in FPG after interval training (IT) intervention was detected compared to a control group. After resistance training (RT) intervention, FPG was significantly lower -6.71 (-12.65,-0.77) mg/dL, Z=2.21, p=0.03, and HbA1c by -0.13 (-0.55, 0.29), p=0.54, compared to the control group. The impact of RT compared to no intervention on 2hPP was not statistically significant (p=0.26). The network meta-analysis did not show statistical significance. Most of the studies presented an unclear risk of bias, and a low and very low-quality of evidence. According to the GRADE criteria, the strength of the body of evidence was low.

**Conclusion:**

Resistance training and IT had demonstrated benefits on glycemic indices, especially on FPG, in a population with prediabetes. Further studies with larger sample sizes and a more robust methodology that compare different types of exercise modalities, frequencies, and durations, are needed to establish a beneficial exercise intervention.

**Systematic review registration:**

https://www.crd.york.ac.uk/prospero/display_record.php?RecordID=370688, identifier CRD42022370688.

## Introduction

Type 2 diabetes (T2D) is a public health problem whose prevalence has increased during recent decades (1, 2), causing an important financial burden on the healthcare system (3). According to the International Diabetes Federation, the global prevalence of diabetes is currently 10.5% (463 million people), and it is projected to rise to 12.2% (783 million people) by 2045 (4). It is predicted that the global economic burden of diabetes will increase from U.S. $1.3 trillion (95% CI 1.3–1.4) in 2015 to $2.5 trillion (2.4–2.6) in 2030 under the past trends (5).

Some risk factors contribute to the development of T2D, including age, overweight/obesity, physical inactivity, family history of diabetes, history of gestational diabetes, and prediabetes (6). Among these factors, prediabetes is the preceding phase for developing diabetes (7), which is characterized by a metabolic state with higher blood glucose levels than normal but below the criteria for a diagnosis of T2D. Prediabetes has been defined by impaired fasting glucose (IFG), impaired glucose tolerance (IGT), and/or increased glycated hemoglobin (HbA1c). People with IGT have increased postprandial blood glucose levels (8), while insulin resistance and beta-cell dysfunction are the main causes of IGT and IFG (9, 10). The American Diabetes Association (ADA) defines prediabetes using IFG defined as fasting plasma glucose (FPG) of 100-125 mg/dL and IGT defined as 2-hour post prandial glucose (2hPP) of 140-200 mg/dL after ingestion of 75 g of oral glucose load and HbA1c based criteria of a level of 5.7% to 6.4% (11). The World Health Organization (WHO), on the other hand, has the same cut-off value for IGT but has a high cut-off value for IFG (FPG 110-125 mg/dL) and did not consider HbA1c (12).

People with prediabetes are at a high risk of developing T2D, especially those who are overweight or obese (13). Lifestyle modifications, including regular physical activity (PA), play a crucial role in preventing the progression to T2D and even reversing prediabetes to normoglycemia (6, 14, 15). Approximately 70% of people with prediabetes will progress to T2D, with an annual progression rate of 5-10% (16, 17).

Several studies have shown the beneficial effects of PA on glycemic control in people with prediabetes (18–20), but there is relatively limited knowledge regarding the effect of structured exercise on glycemic control in this population (21). Moreover, there are few studies comparing the effects of different exercise modalities on glycemic control with an appropriate sample size to generalize the results (22–24). Consequently, it remains unclear which exercise modality and duration is most effective in reducing T2D risk, particularly in individuals with prediabetes.

Among different exercise modalities, aerobic training (AT) and resistance training (RT) improves glycemic control in people with and without prediabetes and T2D through multiple mechanisms (25). These mechanisms include the use of glucose for energy, leading to decreased blood glucose levels over time due to reduced muscle glycogen caused by exercise. Other mechanisms involve enhancing endothelial function; improving pancreatic β-cell functions; enhancing glucose metabolism, reducing visceral adipose tissue (VAT), increasing lean tissue, and increasing the production of glucose transporter type 4 (GLUT-4), which improves insulin sensitivity and enhances glucose uptake, ultimately leading to improved glycemic control (26–31).

Even though the WHO recommends performing moderate-vigorous physical activity (MVPA), accumulating between 150-300 minutes/week, and combining aerobic exercise with resistance training at least 2 days/week (32), few adults comply with these recommendations (33). High-intensity interval training (HIIT) has shown beneficial effects on VO_2_max, insulin resistance (34), and muscle strength (35) in adults and seems to produce positive effects on glycemic control in people with prediabetes (20). Additionally, moderate-intensity interval training (IT) has shown positive results in multiple measures of glycemic variability (36). Furthermore, vigorous-intensity exercise may provide similar or even greater benefits than moderate-intensity exercise for glycemic control in individuals with T2D (37).

Considering these findings, exercise prescription should be a priority in clinical practice to prevent or delay T2D. However, there is a lack of sufficient evidence to determine which exercise modality works best for preventing diabetes in people with prediabetes. Therefore, the purpose of this systematic review with metanalysis is to provide evidence regarding the effect of different exercise modalities on glycemic control in people with prediabetes and to assess which exercise training modality is most effective in improving glycemic control. The research question is as follows: Which exercise modality is more effective in improving glycemic control in people with prediabetes?

## Methods and analysis

### Study protocol and registration

The protocol of this systematic review and meta-analysis was registered on the Prospective Register of Systematic Reviews (PROSPERO) website (https://www.crd.york.ac.uk/prospero/display_record.php?RecordID=370688) and PROSPERO registration number is CRD42022370688.

The protocol of this systematic review was prepared according to the Preferred Reporting Items for Systematic Reviews and Meta-Analyses Protocols (PRISMA-P) statements (38), and the systematic review was equally reported according to PRISMA guidelines (39).

### Search strategy

We performed a systematic literature search of the main databases: MEDLINE (via PubMed), EMBASE, SPORTDiscus (via EBSCO), Web of Science, PEDro, BVS, and the Cochrane Library from the inception to June 2022. There were no restrictions in terms of the language of publication.

Search terms included controlled terms from MeSH in PubMed/MEDLINE and EMtree in EMBASE as well as free text terms. The key search terms were prediabetic state, exercise, and clinical trial. The search strategy was performed in cooperation with an information scientist which is shown in the supplementary information (**Additional File 1**).

Unpublished literature was identified through Clinical Trials (https://clinicaltrials.gov), the Information System on Gray Literature in Europe (Open Gray), Conference Proceedings of the Web of Science and ProQuest Dissertations, and Theses Global. Data from conference proceedings were not included in the review due to the limited information available to carry out the methodological quality assessment.

### Eligibility criteria

#### Inclusion criteria

Our inclusion criteria, framed in terms of PICO (population, intervention, comparator, and outcome) questions are the following:

- Population: Participants at least 18 years old with prediabetes as defined by ADA (11) and/or WHO criteria (12).

- Intervention: We focused on the following exercise training modalities: AT, IT, RT, combined exercise, and no exercise. The definition of each exercise training modality is shown in the supplementary information (**Additional File 2**). Studies were included if the implementation of an exercise program last at least 12 weeks in duration [as most studies agree on long-term interventions of at least 12 weeks to be able to assess changes in many physiological variables, such as anthropometric, biochemical, physical fitness variables (22, 33, 40) and blood glucose (20, 41, 42)]. In addition, HbA1c reflects half of the glucose concentration during the previous 8-12 weeks (43), consequently, a period of 12 weeks or more is necessary to detect changes in HbA1c.

- Comparator: Placebo control/Different exercise modality.

- Outcome: Our primary outcome was the glycemic control therefore we included FPG, HbA1c, and 2hPP measures.

In terms of study design, we only included randomized control trials (RCTs).

#### Exclusion criteria

Study protocols for RCTs and studies focused on pregnant women.

### Study selection

References of the studies identified were imported into EndNote 20 (Clarivate Analytics, Philadelphia, USA) to manage the literature search records. Duplicates were subsequently removed. To ensure the quality of the process, two blinded reviewers (IH- and MB-V) independently screened the title, abstract, or both, in each record for relevance according to the eligibility criteria. Any disagreements or conflicts between the reviewers were resolved through consensus. After this initial screening, all potentially eligible references were evaluated at the full-text level to confirm their eligibility.

### Data extraction and management

We extracted and registered the data about the characteristics of the studies and study results in Microsoft Excel 2019 (Microsoft Corp, Redmond, WA, www.microsoft.com) and Review Manager software (RevMan version 5.4.1, Copenhagen, Denmark: The Nordic Cochrane Centre, the Cochrane Collaboration 2014), respectively. Two reviewers (IH-B and MB-V) independently extracted the data of the characteristics of the studies (e.g., publication year, country, journal title, participants, sample size, diagnostic criteria for prediabetes and study period) and outcomes (FPG, HbA1c, and 2hPP). Since the data for anthropometric variables, fitness level, and health status were not available for all the studies, we did not include them in the present meta-analyses. The reviewers contacted the authors to resolve doubts or questions or request missing or incomplete data. Data were presented as the mean and standard deviation (SD) at the end of the study.

### Risk of bias and quality of evidence assessment

Both reviewers (IH-B and MB-V) independently assessed the risk of bias in the included according to the Cochrane Handbook version 5.1.0 (44). This assessment considered aspects such as adequate sequence generation, allocation concealment, blinding of participants and personnel, incomplete outcome data, selective reporting, and other sources of bias (e.g., extreme baseline imbalance). The methodological quality was classified as having a low, high, or unclear risk of bias. Additionally, another reviewer (AY), with methodological expertise, independently supervised the risk of bias assessment, and any disagreements were resolved by consensus. The quality of the body of evidence was evaluated based on the GRADE criteria (45). We also assessed the potential for publication bias using Egger’s test (46).

### Data analysis

For direct meta-analysis, we analyzed the data using Review Manager software (RevMan version 5.4; Cochrane Collaboration, Copenhagen, Denmark, 2014). Weights and mean difference (95% CI) were determined using random‐effects models. Post-intervention values of FPG, 2hPP, and HbA1c between the control and intervention groups were used to calculate the mean differences. Studies that did not report the post-intervention levels of FPG, 2hPP, and HbA1c were excluded from the final analysis. We converted FPG values from mmol/dL to mg/dL. Further, we used the 95%CI or interquartile range to determine SD in studies that did not report the value of SD. Studies with interventions other than AT, RT, and IT were excluded from the final meta-analysis due to the small number of eligible studies.

We used Cochran’s Q statistic and the inconsistency I^2^ test to assess the heterogenicity between studies. I^2^ values of 25, 50, and 75% are considered indicative of low, moderate, and high heterogeneity, respectively (47).

Since the direct meta-analysis could only provide pairwise comparisons of exercise treatments, a network meta-analysis was used to evaluate the efficacies of the three exercise regimens. To run the network meta-analysis, we used Meta-Insight software (48). Random effect models were chosen based on Cochrane’s Q statistic. We used the mean difference to summarize the effects of the continuous outcome variables.

## Results

### Literature selection

**Figure 1:**
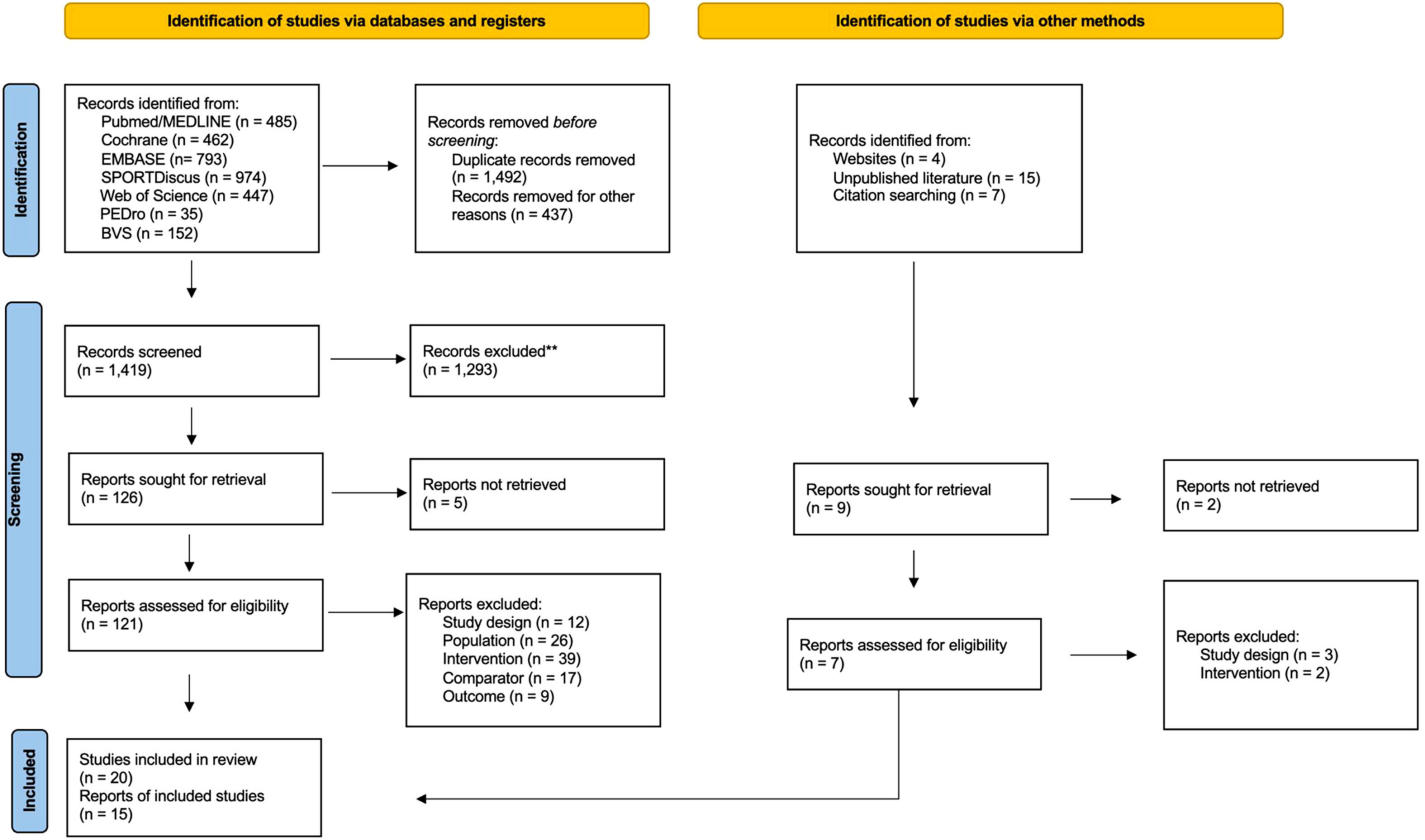
PRISMA Flow diagram of literature search and study selection process.

A total of 1,419 studies were initially identified in this study after duplicates were removed. After reviewing the title and abstract, 126 studies were selected for further review. Among these, 5 studies were excluded because authors did not respond to data requests and 103 did not meet the review eligibility criteria. Finally, 20 studies met our inclusion criteria (20, 22–24, 36, 41, 42, 49–62), of which 15, comprising 775 participants with prediabetes, were combined in the meta-analysis, and 13 in the network meta-analysis. A flow chart of the screening process was presented in accordance with the PRISMA flow diagram of study selection (**Figure 1**).

### Study characteristics of eligible studies

The characteristics of the included studies are illustrated in **Table 1**. The final analysis consisted of 15 studies published between 2012 and 2022, with a total of 775 participants with prediabetes (52% of them were women). In the study by Dai et al., the sex of participants was not reported (23). The total mean (SD) age of the participants in the intervention group was 51.66 (13.36) years and, for the control group, it was 53.1 (12.16) years. The study by Die et al., and Hansen et al., did not report the age of the participants (23, 55).

**Table 1:** Characteristics of included studies.

Author	Year	Country	Diagnostic criteria	Groups	Sample size (% female)	Age (SD)	Duration (frequency)	Type of intervention	Length/session	Intensity	Dropout	Adherence
Alvarez et al.^a^	2012 (49)	Chile	FPG (ADA)	IT Control	12 (100%) 13 (100%)	39.2 (9.5) 40.1 (11.4)	12 weeks (3 days/week) -	Running races Lifestyle advice	20’ -	>85% HR max	nd nd	85% nd
Alvarez et al.^b^	2012 (49)	Chile	FPG (ADA)	RT Control	8 (100%) 13 (100%)	33.9 (9.3) 40.1 (11.4)	12 weeks (2 days/week) -	Free weigh Lifestyle advice	45’ -	VI	nd nd	95% nd
Alvarez et al.^c^	2012 (49)	Chile	FPG (ADA)	IT+RT Control	10 (100%) 13 (100%)	43.3 (8.1) 40.1 (11.4)	12 weeks (5 days/week) -	Combined training Lifestyle advice	20-45’ -	Both combinations	nd nd	74% nd
Burtscher et al.	2009 (50)	Austria	FPG (WHO)	AT Control	18 (55.5%) 18 (55.5%)	59.1 (7.8) 55.8 (5.5)	48 weeks (2.7 days/week) 48 weeks (1.3 days/week)	Jogging, swimming, running, dancing… Lifestyle advice	81’ 85’	Lactate 2-3 mmol/L	nd nd	nd nd
Burtscher et al.^a^	2012 (63)	Austria	FPG (WHO)	AT+RT Control	12 (66.7%) 18 (50%)	57.8 (6.5) 57.8 (7.9)	48 weeks (2 days/week)	Jogging, swimming, strength training… nd	60’	MI (HR or PE)	nd nd	nd nd
Burtscher et al.^b^	2012 (63)	Austria	FPG + IGT (WHO)	AT+RT Control	12 (66.7%) 18 (50%)	54.0 (8.0) 57.6 (5.8)	48 weeks (2 days/week)	Jogging, swimming, strength training… nd	60’	MI (HR or PE)	nd nd	nd nd
Chen et al.	2021 (51)	China	FPG (ADA) IGT (ADA) HbA1c (ADA)	AT RT Control	83 (71.1%) 82 (63.4%) 83 (60.2%)	60.9 (5.7) 59.9 (5.9) 60.7 (5.8)	12 and 24 months (3 days/week) 12 and 24 months (3 days/week)	Aerobic dance Elastic bands Maintain usual habits	60’ 50’	60-70% HRmax -	18.3%; 26.8% 18.1% 30.1% 14.5%; 33.7%	81.7%; 73.2% 81.9%; 69.9% 85.5%; 66.3%
Dai et al. ^a^	2019 (23)	China	ADA	AT Control	41 (nd) 45 (nd)	55-75 (nd) 55-75 (nd)	96 weeks (3 days/week)	Dancing Lifestyle advice	60’	60-70% HR max	17.1% 22.2%	nd nd
Dai et al. ^b^	2019 (23)	China	ADA	RT Control	43 (nd) 45 (nd)	55-75 (nd) 55-75 (nd)	96 weeks (3 days/week)	Leg and chest press, pull downs… Lifestyle advice	60’	60-80% 1RM	27.9% 22.2%	nd nd
Dai et al. ^c^	2019 (23)	China	ADA	AT+RT Control	43 (nd) 45 (nd)	55-75 (nd) 55-75 (nd)	96 weeks (3 days/week)	Combined training Lifestyle advice	30’+30’	Both combinations	13.9% 22.2%	nd nd
Desch et al.	2010 (23)	Germany	IGT FPG	AT Control	14 (21.4) 12(33.3)	62.3 (6.2) 62.3 (6.5)	6 months (1 o 2 days/week)	Bicycle ergometer	90’-120’	75% HRmax	nd	nd
Færch et al.	2021 (36)	Denmark	HbA1c (ADA)	IT Control	30 (50%) 30 (60%)	57.8 (9.9) 57.2 (9.9)	13 weeks (5 days/week)	Walking, cycling, running… Lifestyle advice	30’	≥75%/≤60% HR max	nd nd	93% nd
Fritz et al.^**^	2013 (36)	Stockholm	IGT (8.9-12.1 mmol/L)	AT Control	14 (64.3%) 21 (52.4%)	59.1 (6.2) 61.8 (3.4)	16 weeks (nd)	Nordic walking nd	300’	MI (HR or PE)	nd nd	>80% nd
Gidlund et al.^a^	2016 (22)	Finland	FPG (ADA) IGT (ADA) Findrisk (>12)	AT Control	20 (0%) 17 (0%)	54 (6.2) 54(6.9)	12 weeks (3 days/week)	Nordic walking Lifestyle advice	60’	55% to 75% HRR	nd nd	nd nd
Gidlund et al.^b^	2016 (22)	Finland	FPG (ADA) IGT (ADA) Findrisk (>12)	RT Control	18 (0%) 17 (0%)	56 (5.6) 54(6.9)	12 weeks (3 days/week)	Leg and bench press, leg extension… Lifestyle advice	60’	50% to 85% 5RM	nd nd	nd nd
Gilbertson et al.	2019 (54)	EEUU	ADA	AT IT	12 (41.4%) 17 (58.6%)	50.8 (4.4) 45.7 (4.4)	16 weeks (3 days/week) 16 weeks (3 days/week)	Walk and run-on treadmill Walk and run-on treadmill	30-60’ 45’	45-55% HRR 10 bpm of HRmax/PE^1^	38.0% 10.3%	89.6% 91.7%
Hansen et al.	2012 (55)	Norway	IGT (WHO)	RT RT	9 (77.8%) 9 (77.8%)	33-69 (nd) 33-69 (nd)	16 weeks (3 days/week) 16 weeks (3 days/week)	Leg and chest press, pull downs… Leg and chest press, pull downs…	nd nd	60%-85% 1RM 45%-65% 1RM	nd nd	nd nd
Herzig et al.	2014 (56)	Finland	FPG or IGT (WHO)	AT Control	33 (72.7%) 35 (74.3%)	58.1 (9.9) 59.5 (10.8)	12 weeks (3 days/week)	Indoor sports nd	60’	MI (walk at 3-4 km/h)	13.1% 12.5%	67% nd
Liao et al.	2015 (41)	nd	FPG (ADA)	AT Control	60 (47%) 60 (41.7%)	42.4 (5.8) 44.1 (6.6)	12 weeks (5 days/week)	Jogging or brisk walking Lifestyle advice	30’	MI	13.3% 6.7%	nd nd
Liu et al.^a^	2013 (57)	China	IGT (7.8-10 mmol/L)	AT Control	20 (nd) 21 (nd)	49.8 (4.8) 49.8 (4.8)	24 weeks (4 days/week)	Walk and run-on treadmill nd	60’	60%-70% HRmax	nd	nd
Liu et al.^b^	2013 (57)	China	IGT (7.8-10 mmol/L)	AT+RT Control	20 (nd) 21 (nd)	49.8 (4.8) 49.8 (4.8)	24 weeks (4 days/week)	Walk/upper arm, chest and waist … nd	50’ (30’ RT+20’AT)	nd	nd	nd
Malin et al.	2012 (58)	EEUU	IGT (ADA)	AT+RT Control	8 (62.5%) 8 (75.0%)	45.4 (8.0) 49.8 (10.9)	12 weeks (3 days/week)	Cycling, free weigh Placebo	60-75’	70% HR/70% of 1RM	nd nd	nd nd
RezkAllah et al.^a^	2019 (20)	Egypt	FPG (ADA)	IT Control	20 (45%) 20 (40%)	31.8 (5.3) 35.9 (5.8)	12 weeks (3 days/week)	Uphill running on treadmill Lifestyle advice	25’	90% HRmax	nd nd	nd nd
RezkAllah et al.^b^	2019 (20)	Egypt	ADA	IT Control	20 (50%) 20 (40%)	31.0 (5.3) 35.9 (5.8)	12 weeks (3 days/week)	Uphill running on treadmill Lifestyle advice	45’	90% HRmax	nd nd	nd nd
Rowan et al.	2017 (59)	Canada	HbA1c (ADA)	IT+RT AT+RT	10 (33.3%) 11 (63.6%)	47.7 (6.9) 53.6 (8.2)	16.6 weeks (3 days/week)	Running on treadmill, push-ups, squats Running on treadmill, push-ups, squats	38’ 38’	90% HRR 60-70% HRR	20% 0%	80% 100%
Slentz et al. ^a **^	2016 (60)	EEUU	FPG (5.28 -6.94 mmol/l)	AT Control	40 (57.5%) 37 (54.0%)	61.4 (7.1) 57.6 (8.1)	24 Weeks (8.6 milles/week) 24 Weeks (8.6 milles/week)	Cardiovascular machines Clinical lifestyle	≤ 60’	50% VO_2_ reserve 50% VO_2_ reserve	18% 14%	82% 86%
Slentz et al. ^b **^	2016 (60)	EEUU	FPG (5.28 -6.94 mmol/l)	AT Control	38 (60.5%) 37 (54.0%)	60.4 (7.0) 57.6 (8.1)	24 Weeks (13.8 milles/week) 24 Weeks (8.6 milles/week)	Cardiovascular machines Clinical lifestyle	≤ 60’	50% VO_2_ reserve 50% VO_2_ reserve	15% 14%	85% 86%
Slentz et al. ^c **^	2016 (60)	EEUU	FPG (5.28 -6.94 mmol/l)	AT Control	35 (62.8%) 37 (54.0%)	56.9 (7.8) 57.6 (8.1)	24 Weeks (13.8 milles/week) 24 Weeks (8.6 milles/week)	Cardiovascular machines Clinical lifestyle*	≤ 60’	75% VO_2_ reserve 50% VO_2_ reserve	15% 14%	85% 86%
Venojärvi et al.	2013 (61)	Finland	FPG (ADA) IGT (ADA)	AT RT CG	48 (0) 49 (0) 47 (0)	55 (6.2) 54 (6.1) 54 (7.2)	12 weeks (3 days/week) 12 weeks (3 days/week)	Nordic walking Strength and power exercise Lifestyle advice	60’ 60’	55-75% HRmax 50-85% 1RM	18.7% 26.5% 14.9%	81.3% 73.5% 85.1%
Viskochil et al.	2017 (61)	EEUU	IGT (ADA)	AT+RT Control	9 (55.5%) 8 (75.0%)	46.2 (2.6) 49.8 (3.9)	12 weeks (3 days/week)	Cycling, free weigh Placebo	60-75’	65% VO_2_ max./60%-70% 1RM -	nd nd	nd nd
Yan et al.^a^	2019 (62)	China	IGT (ADA)	AT Control	35 (71.4%) 35 (57.1%)	64.2 (5.7) 60.3 (7.6)	48 weeks (3 days/week)	Aerobic dancing Lifestyle advice	50’	60-70% HRmax	11.4% 5.7%	88.6% 94.3%
Yan et al.^b^	2019 (62)	China	IGT (ADA)	RT Control	35 (57.1%) 35 (57.1%)	62.1 (8.1) 60.3 (7.6)	48 weeks (3 days/week)	Leg and chest press, pull downs… Lifestyle advice	60’	60% 1RM	17.2% 5.7%	82.8% 94.3%
Yuan et al.^a^	2019 (62)	China	FPG (ADA) IGT (ADA) HbA1c (ADA)	AT Control	83 (71.1%) 83 (60.2%)	59.9 (5.9) 60.7 (5.8)	24 weeks (3 days/week)	Aerobic exercises Lifestyle advice	60’	60-70% HRmax	12% 12%	88% 88%
Yuan et al.^b^	2019 (62)	China	FPG (ADA) IGT (ADA) HbA1c (ADA)	RT Control	82 (63.4%) 83 (60.2%)	60.9 (5.7) 60.7 (5.8)	24 weeks (3 days/week)	Leg and chest press, pull downs… Lifestyle advice	50’	60% 1RM	13.4% 12%	86.6% 88%

FPG, fasting plasma glucose; HbA1c, glycated hemoglobin; 2hPP, 2-hour postprandial; AT, Aerobic training; RT, Resistance training; IT, Interval training; IT+RT, a combination of IT and RT; AT+RT, a combination of AT and RT; VI, vigorous intensity; MI, moderate intensity; HRmax, maximum heart rate; HRR, heart rate reserve; VO_2_ max., maximum consumption of oxygen; 1RM, one repetition maximum. 5RM, five repetitions maximum; PA, physical activity; PE, perceived exertion._2_ max.: maximum consumption of oxygen; 1RM: one repetition maximum. 5RM: five repetitions maximum; PA: physical activity; PE: perceived exertion.

Vigorous intensity can be determined by HR ≥85% HRmax. or by VO_2_ max. ≥60% (64–67).

^1^The Borg Rating of Perceived Exertion scale is a tool for measuring an individual’s effort and exertion, breathlessness, and fatigue during exercise practice (68), a perceived exertion of 19-20 on the Borg scale corresponds to “extremely hard and maximal exertion” respectively (69).

*Clinical lifestyle: Diet + cardiovascular machines.

**This study was not included in the meta-analysis.nd = not determined.

The included studies used different exercise modalities, with a wide variation in session duration ranging from 20 to 85 minutes. These exercise modalities can be divided into three main categories: AT, RT, and IT. The duration of the studies ranged between 12 to 96 weeks.

Studies with more than one mode of exercise intervention were analyzed in different meta-analysis groups with the same control group. Studies that consisted of interventions with a combination of two exercise modalities (e.g., IT+RT vs. control) and studies with diet or exercise interventions as control groups were excluded due to the impossibility of comparison with other studies.

### Risk of bias and quality of evidence

**Figure 2:**
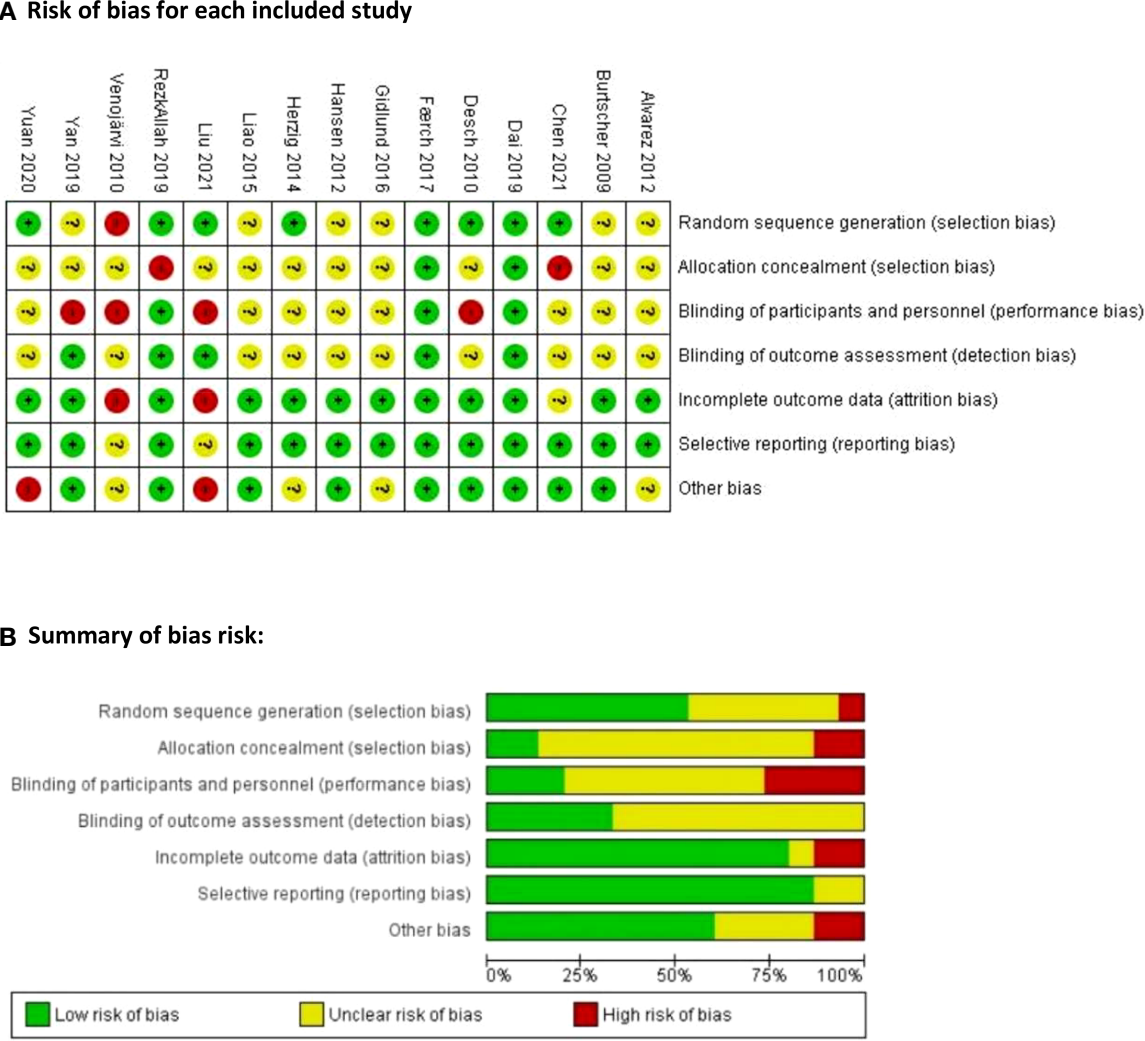
Risk of bias assessment for the included studies. **(A)** Risk of bias for each included study: “+” represents low risk of bias; “–” represents high risk of bias; and “?” represents unclear risk of bias **(B)** Summary of bias risk:.

The quality of the included studies was assessed as moderate (**Figure 2**). Blinding of participants and personnel was not applicable in most studies due to the nature of the intervention, but unfortunately, they did not report any description of it. Random sequence generation, allocation concealment, and blinding of outcome assessors presented an unclear risk of bias in several studies. However, it was noted that most of the studies had a low risk of attrition and reporting bias. According to the GRADE criteria, the strength of the body of evidence was determined to be low. As only RCTs were included, the strength of the body of evidence was initially considered to be high in terms of the primary outcomes (45). However, due to the small sample size and the risk of bias observed in a considerable proportion of studies, the rating was downgraded to moderate.

### Description of exercise modalities

#### Direct meta-analysis

##### Fasting plasma glucose (FPG)

**Figure 3:**
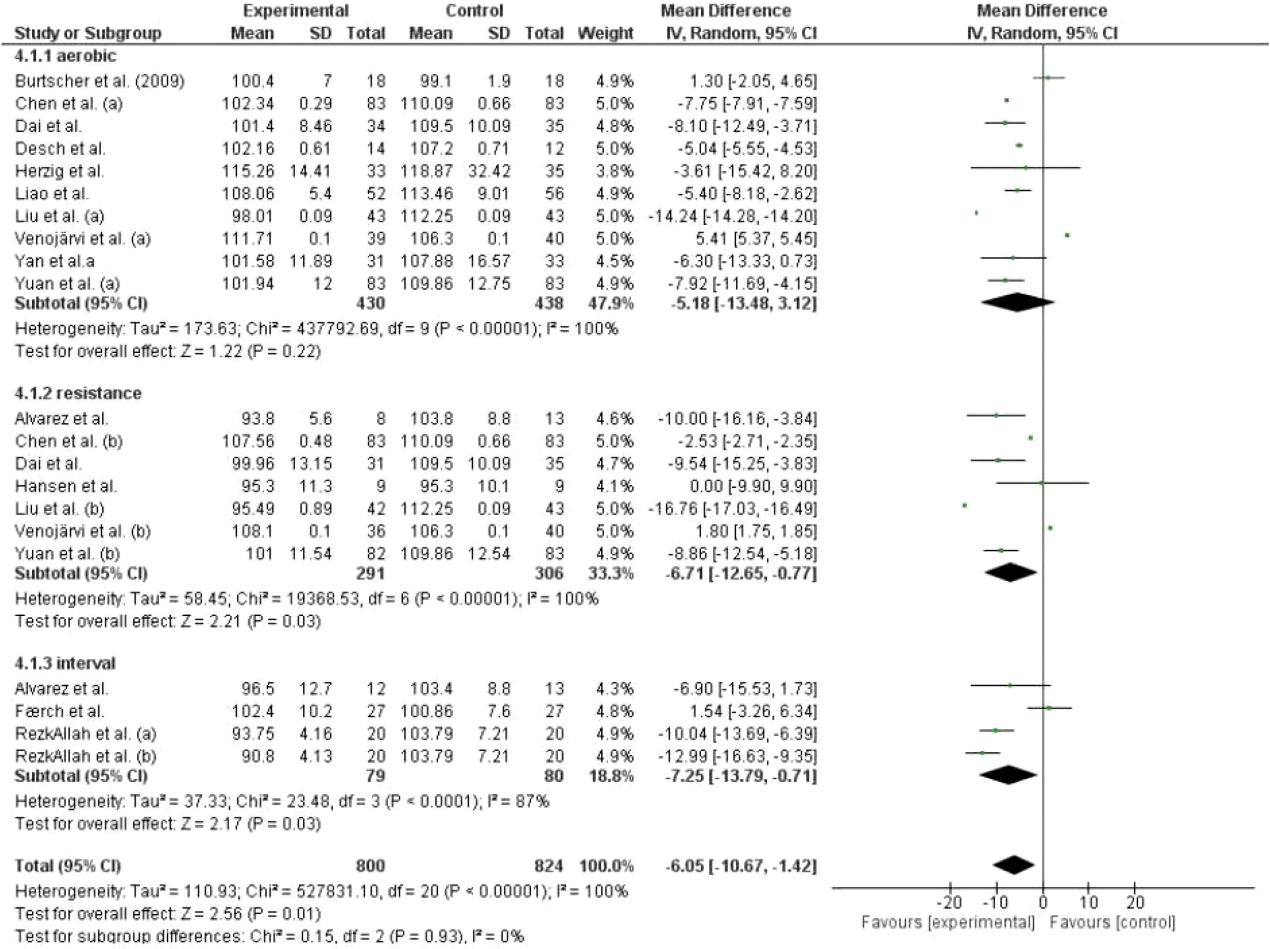
Effect of different exercise modalities versus control (no intervention) on FPG.

Meta-analysis results did not show a statistically significant reduction in FPG after different types of AT compared to the control group (p=0.22). However, studies evaluating RT showed a significant reduction of -6.71 in FPG levels compared to the control group (p=0.03). Similarly, IT resulted in a significant reduction of -7.25 in FPG levels (p=0.03). Detailed information is shown in **Figure 3**.

##### Glycated hemoglobin (HbA1c)

**Figure 4:**
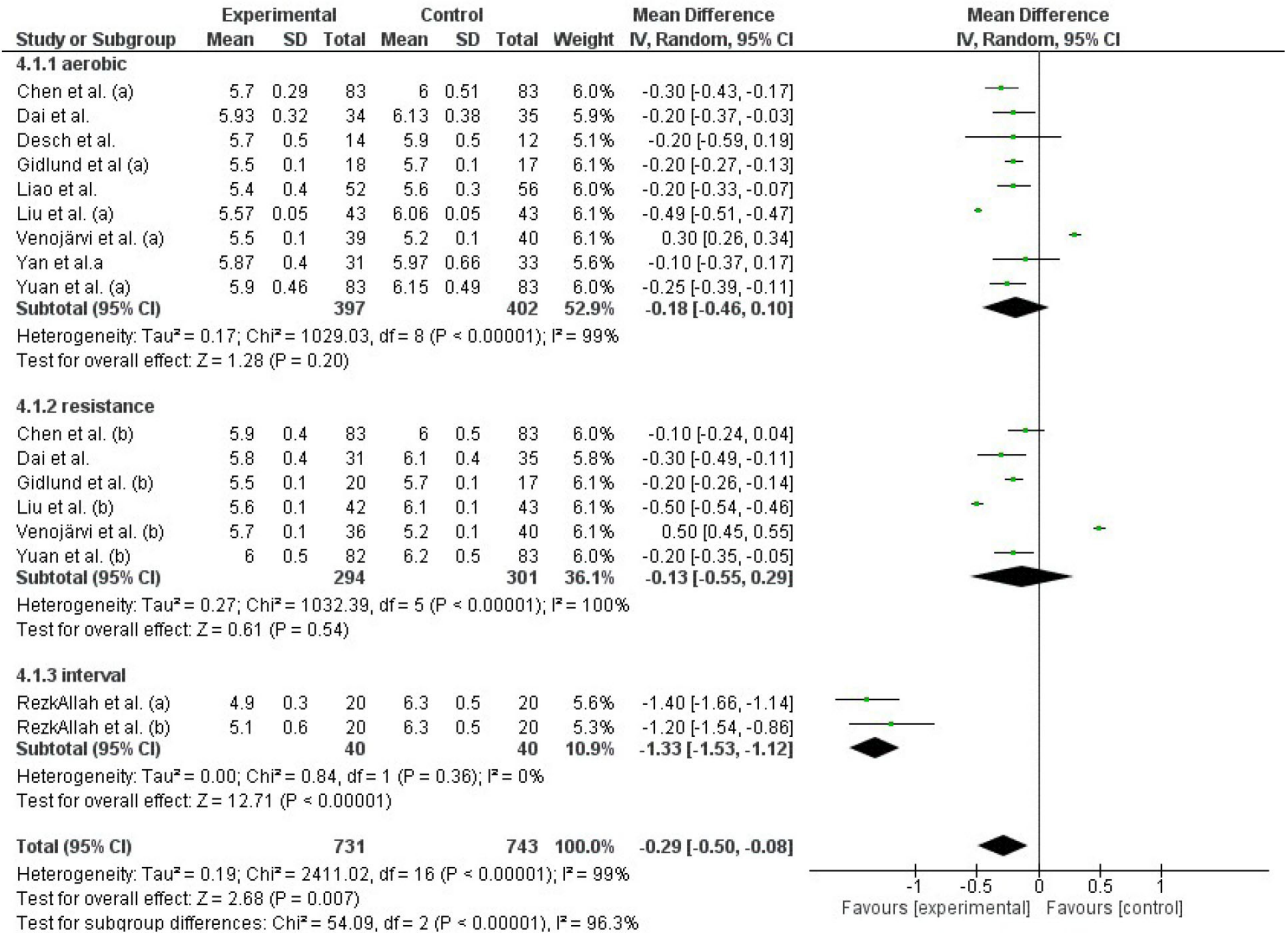
Effect of different exercise modalities versus control (no intervention) on HbA1c.

Both, AT and RT as exercise modalities (**Figure 4**) did not show a significant effect on HbA1c levels (p>0.05). However, IT resulted in a significant reduction in HbA1c levels compared to the control group (mean difference [95%CI]: -1.33 [-1.53,-1.12], p<0.0001).

##### 2-Hour postprandial glucose (2hPP)

**Figure 5:**
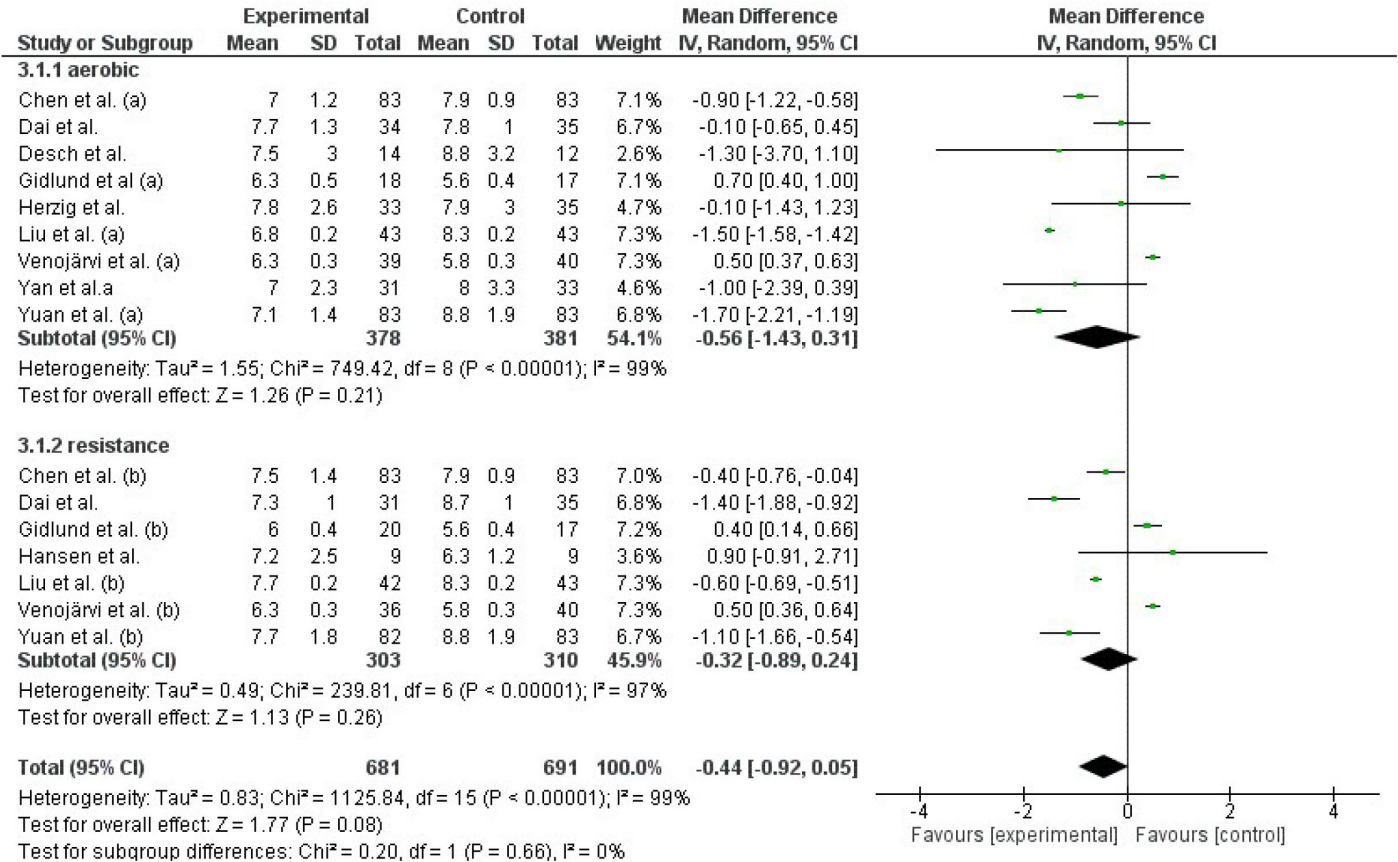
Effect of different exercise modalities versus control (no intervention) on 2hPP.

Results from the studies that used AT and RT as interventions showed no significant reduction in 2hPP levels (**Figure 5**).

In total AT, RT and IT had a significant effect on reducing FPG levels (Z=2.56, p=0.01). Similarly, the general effect of AT, RT, and IT was statistically significant in reducing HbA1c levels (Z=2.68, p=0.008). However, this effect was not observed in studies that evaluated 2hPP levels using AT and RT as interventions (Z=1.77, p=0.08).

### Network meta-analysis

**Figure 6:**
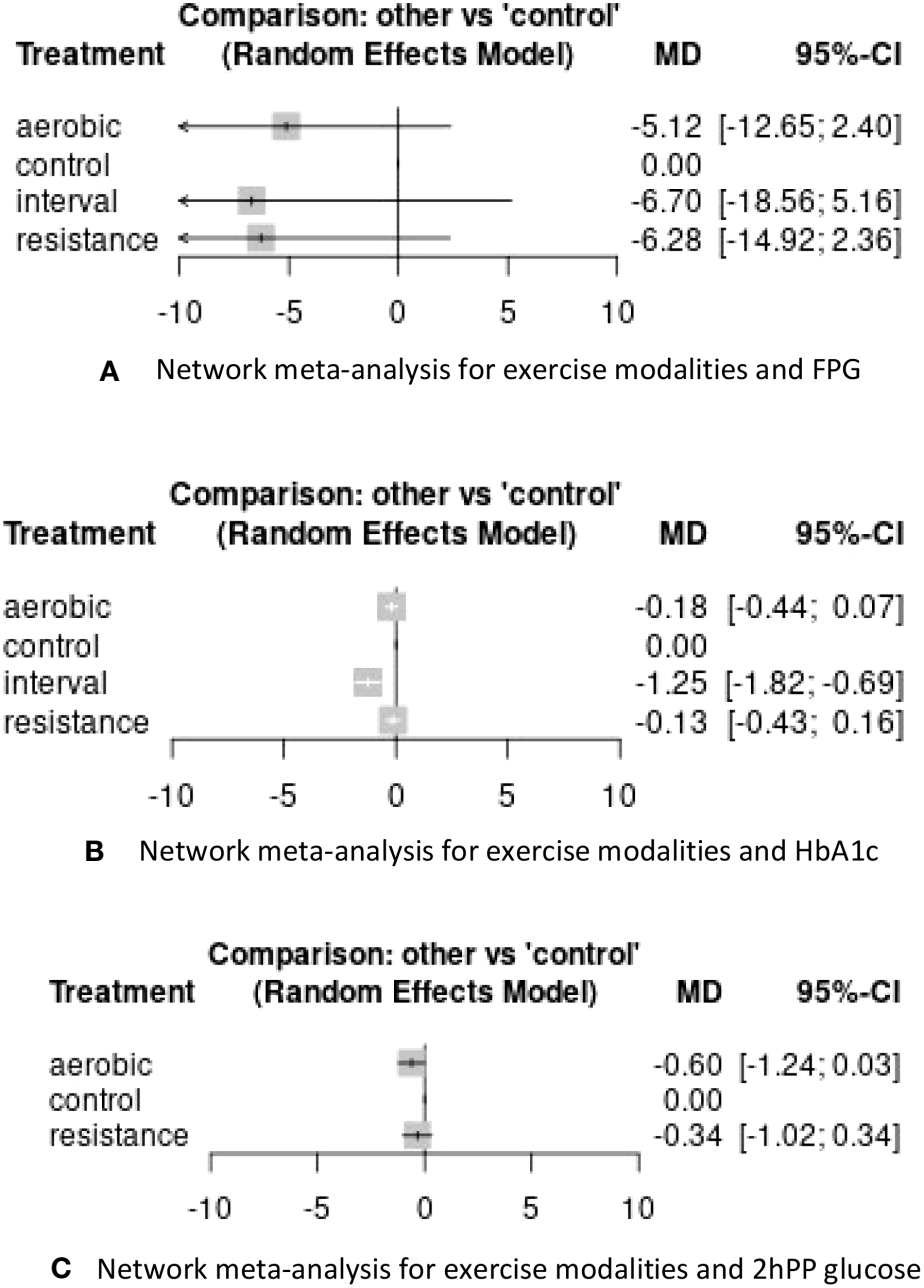
Results of network meta-analysis for exercise modalities and glycemic control variables. **(A)** Network meta-analysis for exercise modalities and FPG **(B)** Network meta-analysis for exercise modalities and HbA1c **(C)** Network meta-analysis for exercise modalities and 2hPP glucose.

Thirteen studies were included in the network meta-analysis. When comparing different types of physical activity to no exercise, all exercise modalities showed a decrease in glycemic indices (**Figure 6**). The effect of exercise modalities on lowering FPG and HbA1c was higher for IT compared to other exercise modalities (Mean Difference: -6.70, 95%CI: -18.56, 5.16 for FPG, Mean Difference: -1.25, 95%CI: -1.82,-0.69 for HbA1c). This effect was statistically significant for the reduction of HbA1c. However, for 2hPP levels, the studies included in the network meta-analysis involved AT and RT. Nevertheless, both exercise modalities did not have a notable effect in reducing 2hPP levels in participants with prediabetes (**Figure 6C**).

**Figure 7:**
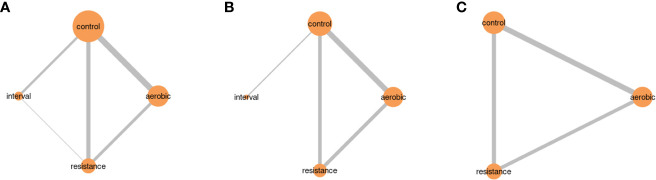
Network plots for studies included in network meta-analysis. **(A)** Exercise modalities and FPG **(B)** Exercise modalities and HbA1c **(C)** Exercise modalities and 2hPP glucose.

The size of the nodes is related to the number of participants in that intervention type, and the thickness of the lines connecting interventions is linked to the number of studies for that comparison (**Figure 7**).

### Inconsistency between direct and indirect comparisons

The assessment of inconsistency between direct and indirect comparison (**Table 2**) revealed that no studies had inconsistencies (p value> 0.05).

**Table 2:** Results of the consistency test.

Comparison	No. Studies	NMA	Direct	Indirect	Difference	Diff_95CI_lower	Diff_95CI_upper	P
aerobic:control	10	-5.12457	-5.18343	-3.65091	-1.53253	-40.7095	37.6444	0.938886
aerobic:interval	0	1.574538	NA	1.574538	NA	NA	NA	NA
aerobic:resistance	5	1.159878	0.648912	2.800956	-2.15204	-24.1807	19.8766	0.848154
interval:control	4	-6.69911	-7.23204	1.854081	-9.08612	-59.5678	41.39555	0.72426
resistance:control	7	-6.28445	-6.63509	-3.65317	-2.98192	-29.8052	23.84132	0.827517
interval:resistance	1	-0.41466	2.7	-1.78363	4.483629	-25.7406	34.70788	0.771241

NA, not available.

## Discussion

The main findings of the present meta-analysis indicate that IT and RT are useful interventions to control FPG levels in people with prediabetes. In contrast, AT does not show significant differences in FPG levels compared to a control group, and neither does RT in terms of HbA1c or 2hPP glucose levels. Additionally, the network meta-analysis shows that any type of PA in comparison with no exercise achieves lower glycemic indices, and IT shows differences with all other modalities for HbA1c. However, no significant differences were found between AT and RT in any glycemic indexes.

A previous meta-analysis performed with a population with T2D and prediabetes compared HIIT with moderate-intensity continuous training, and found no significant differences in FPG or HbA1c (70). In that meta-analysis, only one study include a population with prediabetes (71), and no differences were also found as well. Another meta-analysis showed that both AT and RT led to reductions in HbA1c compared to a control group (72). In our meta-analysis, we only identified three studies that compared AT with RT, but no significant differences were found. This could be due to the small sample size, or the limited number of studies included in the analysis.

Interestingly, some evidence suggests that AT appeared to be more effective in isolated IGT as it conferred benefits in 2hPP (73). However, a previous systematic review with diabetic patients concluded that, despite differences in some glycemic control reaching statistical significance in favor of AT, there was no evidence that these differences were of clinical importance or had an impact on cardiovascular risk markers or safety (74). On the other hand, there are contradictions in the role of RT on glycemic control (75–77). In a meta-analysis with the T2D population, RT showed reductions in HbA1c, but there were no correlations between RT intensity, duration, frequency, and changes in HbA1c levels (76). Another meta-analysis with the T2D population observed greater reductions in HbA1c when RT was performed at a moderate-vigorous intensity compared to light intensity, indicating that the training component with the greatest effect on HbA1c is intensity, rather than frequency or duration (77, 78). Another study conducted with an adult population with T2D showed that RT was more effective than AT in HbA1c control (75). However, the small sample size of the study (n=20) and the short duration of the intervention (only 10 weeks) could limit the changes in HbA1c. In contrast, a recent meta-analysis with a prediabetic population that compared AT with RT or a combination of both, concluded that all modalities exerted beneficial effects, but AT or a combination of AT and RT provided better glycemic control than RT alone (72). Moreover, there is evidence suggesting that a combination of AT+RT could provide greater benefits in glycemic control than both modalities separately (79).

One of the possible explanations for the inconsistency of the results obtained in the literature may be due to small sample sizes and interventions not being implemented in a controlled, supervised, and systematic manner. For instance, Yan et al. (2019) found an improvement in HbA1c levels with AT after 12 months of intervention compared to RT or the control group, with 35 participants in each group (24). Similarly, Dai et al. (2019) observed a reduction in FPG, HbA1c, and 2hPP in all intervention groups (AT, RT, and AT+RT) compared to the control group, with a similar sample size. In this study, the group that showed the most significant reduction in FPG levels was AT+RT, followed by RT and AT, with a TD2 incidence reduction of 74% in AT+RT, 65% in RT, and 72% in AT (23). By contrast, Yuan et al. (2019) compared AT with RT and a control group and found no differences between AT and RT groups in glycemic control to FPG, 2hPP, or HbA1C (62). However, significant improvements were observed in all three glycemic control variables when comparing AT or RT separately with the control group. In this study, each group had a sample size of 80 participants, and the exercise sessions were well-detailed and supervised.

Rezkallah and Takla (2019) compared an intervention with low versus high volume HIIT with a control group (n=20 in each group). Both interventions improved FPG and HbA1c compared to the control group, and high-volume HIIT showed greater reductions in HbA1C (20). The remarkable aspect of this article is the high intensity of the intervention and the detailed description of the sessions, which ensures a systematization of the intervention. This finding is consistent with other studies that have suggested that the reduction in HbA1C after HIIT is the result of a lowering of hepatic endogenous glucose production (80).

Based on the data of the included studies in this systematic review with meta-analysis, physical activity has a positive effect on the parameters of glycemic control. However, there is insufficient evidence to determine which type of exercise, intensity, duration, and frequency is most beneficial for glycemic control in people with prediabetes. Further research with methodological rigor and larger sample sizes, such as the GLYCEX study (81), should be conducted to provide better levels of evidence to determine which exercise modality is most effective for glycemic control in people with prediabetes.

Among the strengths of this study, we would like to highlight the registration of protocol in PROSPERO, the adoption of state-of-the-art analytical methods, and a comprehensive search strategy that enabled the inclusion of a large number of studies. An extensive search for relevant studies was conducted in literature sources, grey literature, and reference lists of eligible articles. When necessary, the authors of potentially eligible studies were contacted to obtain additional data for meta-analyses. Moreover, followed the Preferred Reporting Items for Systematic Reviews and Meta-Analyses Protocols (PRISMA-P) statements.

However, some limitations should also be acknowledged. Firstly, the control group intervention was not described in detail in most of the studies, possibly leading to an underestimation of the beneficial effects of different exercise modalities when compared to an active control group. Secondly, there is currently no consensus regarding the diagnostic criteria for prediabetes. The ADA defined prediabetes as an FPG between 100 to 125 mg/dL (82), while the WHO define it as an FPG of 110 to 125 mg/dL (83, 84). Moreover, the cut-off levels for HbA1c vary across different guidelines (85, 86). As a result, different studies included subjects with IFG or IGT or both, which could have acted as potential confounders that influenced the results of the meta-analysis. Thirdly, it is possible that this review did not include all relevant publications due to insufficient information, unavailability of authors, or unanswered communication attempts. Fourthly, the variations in duration, frequency (2-5 days/week), length of sessions 20-90 minutes) and intensity (45-90% HR max) among the included studies could limit the comparison of intervention effects. Furthermore, different types of activities were included for AT interventions, such as running, brisk walking, aerobic dancing, nordic walking, and cardiovascular machines, among others. Moreover, in some studies, the exercise intensity was not well defined or was described in a vague manner (41, 50, 56, 57, 60). Lastly, most of the studies showed methodological limitations, such as small sample sizes (8-21 participants per group) and lack of clear information in some data.

## Conclusion

This review suggests that exercise interventions could be effective in individuals with prediabetes to reduce the risk of developing T2D. However, these results should be taken with caution as the main variable of assessment in this meta-analysis was glycemic control Engaging in any type of physical exercise leads to improved glycemic control compared to no exercise. Our findings showed that AT was not effective in glycemic control, while RT and IT have demonstrated significant benefits, especially in FPG levels, in individuals with prediabetes compared to a control group. Further studies with larger sample sizes and including control groups are needed to determine which exercise modality, frequency, and duration are needed to reverse prediabetes status and prevent the progression to T2D.

## Data availability statement

The original contributions presented in the study are included in the article/**Supplementary Material**. Further inquiries can be directed to the corresponding author.

## Author contributions

MB-V, IH-B, and AY contributed to the conception and design of the study. MB-V, NM, AG-P, IH-B, and AY contributed to data acquisition. NG-C and AG-P provided expertise on the topic of the review. IR-C provided expertise in systematic review and meta-analysis methodology. NM and AY performed the statistical analysis. MB-V, NM, AG-P, and AY drafted the manuscript. All authors contributed to the interpretation of the data and approved the final manuscript.

## References

[1] N. C. D. Risk Factor Collaboration . Worldwide trends in diabetes since 1980: a pooled analysis of 751 population-based studies with 4.4 million participants. Lancet (2016) 387(10027):1513–30. doi: 10.1016/S0140-6736(16)00618-8 PMC5081106 27061677

[2] Chamnan P Simmons RK Forouhi NG Luben RN Khaw KT Wareham NJ . Incidence of type 2 diabetes using proposed HbA1c diagnostic criteria in the european prospective investigation of cancer-norfolk cohort: implications for preventive strategies. Diabetes Care (2011) 34(4):950–6. doi: 10.2337/dc09-2326 PMC3064056 20622160

[3] Standl E Khunti K Hansen TB Schnell O . The global epidemics of diabetes in the 21st century: Current situation and perspectives. Eur J Prev Cardiol (2019) 26(2_suppl):7–14. doi: 10.1177/2047487319881021 31766915

[4] International Diabetes Federation . IDF Diabetes Atlas. 10th ed. Brussels, Belgium: International Diabetes Federation (2021). Available online: https://diabetesatlas.org/atlas/tenth-edition/.

[5] Bommer C Sagalova V Heesemann E Manne-Goehler J Atun R Barnighausen T . Global economic burden of diabetes in adults: projections from 2015 to 2030. Diabetes Care (2018) 41(5):963–70. doi: 10.2337/dc17-1962 29475843

[6] Tabak AG Herder C Rathmann W Brunner EJ Kivimaki M . Prediabetes: a high-risk state for diabetes development. Lancet (2012) 379(9833):2279–90. doi: 10.1016/S0140-6736(12)60283-9 PMC3891203 22683128

[7] Khetan AK Rajagopalan S . Prediabetes. Can J Cardiol (2018) 34(5):615–23. doi: 10.1016/j.cjca.2017.12.030 29731022

[8] Bock G Dalla Man C Campioni M Chittilapilly E Basu R Toffolo G . Pathogenesis of pre-diabetes: mechanisms of fasting and postprandial hyperglycemia in people with impaired fasting glucose and/or impaired glucose tolerance. Diabetes (2006) 55(12):3536–49. doi: 10.2337/db06-0319 17130502

[9] Carnevale Schianca GP Rossi A Sainaghi PP Maduli E Bartoli E . The significance of impaired fasting glucose versus impaired glucose tolerance: importance of insulin secretion and resistance. Diabetes Care (2003) 26(5):1333–7. doi: 10.2337/diacare.26.5.1333 12716784

[10] Kanat M Norton L Winnier D Jenkinson C DeFronzo RA Abdul-Ghani MA . Impaired early- but not late-phase insulin secretion in subjects with impaired fasting glucose. Acta Diabetol (2011) 48(3):209–17. doi: 10.1007/s00592-011-0285-x 21553243

[11] American Diabetes Association . Classification and diagnosis of diabetes: standards of medical care in diabetes-2022. Diabetes Care (2022) 45(Suppl 1):S17–38. doi: 10.2337/dc22-S002 34964875

[12] World Health Organization . Definition and diagnosis of diabetes mellitus and intermediate hyperglycemia: report of a WHO/IDF consultation. Geneva: World Health Organization (2006).

[13] Nathan DM Davidson MB DeFronzo RA Heine RJ Henry RR Pratley R . Impaired fasting glucose and impaired glucose tolerance: implications for care. Diabetes Care (2007) 30(3):753–9. doi: 10.2337/dc07-9920 17327355

[14] Kivimaki M Tabak AG . Does addressing prediabetes help to improve population health? Lancet Diabetes Endocrinol (2018) 6(5):354–6. doi: 10.1016/S2213-8587(18)30030-5 29500120

[15] Zhang X Gregg EW Williamson DF Barker LE Thomas W Bullard KM . A1C level and future risk of diabetes: a systematic review. Diabetes Care (2010) 33(7):1665–73. doi: 10.2337/dc09-1939 PMC2890379 20587727

[16] Hostalek U . Global epidemiology of prediabetes - present and future perspectives. Clin Diabetes Endocrinol (2019) 5:5. doi: 10.1186/s40842-019-0080-0 31086677 PMC6507173

[17] Bennasar-Veny M Fresneda S Lopez-Gonzalez A Busquets-Cortes C Aguilo A Yanez AM . Lifestyle and progression to type 2 diabetes in a cohort of workers with prediabetes. Nutrients (2020) 12(5):1538. doi: 10.3390/nu12051538 32466178 PMC7284825

[18] Boniol M Dragomir M Autier P Boyle P . Physical activity and change in fasting glucose and HbA1c: a quantitative meta-analysis of randomized trials. Acta Diabetol (2017) 54(11):983–91. doi: 10.1007/s00592-017-1037-3 28840356

[19] Jadhav RA Hazari A Monterio A Kumar S Maiya AG . Effect of physical activity intervention in prediabetes: A systematic review with meta-analysis. J Phys Act Health (2017) 14(9):745–55. doi: 10.1123/jpah.2016-0632 28422560

[20] RezkAllah SS Takla MK . Effects of different dosages of interval training on glycemic control in people with prediabetes: A randomized controlled trial. Diabetes Spectr (2019) 32(2):125–31. doi: 10.2337/ds18-0024 PMC6528388 31168283

[21] Rowan CP Riddell MC Jamnik VK . The prediabetes detection and physical activity intervention delivery (PRE-PAID) program. Can J Diabetes (2013) 37(6):415–9. doi: 10.1016/j.jcjd.2013.09.003 24321723

[22] Gidlund EK von Walden F Venojarvi M Riserus U Heinonen OJ Norrbom J . Humanin skeletal muscle protein levels increase after resistance training in men with impaired glucose metabolism. Physiol Rep (2016) 4(23):e13063. doi: 10.14814/phy2.13063 27923980 PMC5357820

[23] Dai X Zhai L Chen Q Miller JD Lu L Hsue C . Two-year-supervised resistance training prevented diabetes incidence in people with prediabetes: A randomised control trial. Diabetes Metab Res Rev (2019) 35(5):e3143. doi: 10.1002/dmrr.3143 30768758

[24] Yan J Dai X Feng J Yuan X Li J Yang L . Effect of 12-Month resistance training on changes in abdominal adipose tissue and metabolic variables in patients with prediabetes: A randomized controlled trial. J Diabetes Res (2019) 2019:8469739. doi: 10.1155/2019/8469739 31737686 PMC6815994

[25] Adams OP . The impact of brief high-intensity exercise on blood glucose levels. Diabetes Metab Syndr Obes (2013) 6:113–22. doi: 10.2147/DMSO.S29222 PMC3587394 23467903

[26] Asano RY Sales MM Browne RA Moraes JF Coelho Junior HJ Moraes MR . Acute effects of physical exercise in type 2 diabetes: A review. World J Diabetes (2014) 5(5):659–65. doi: 10.4239/wjd.v5.i5.659 PMC4138589 25317243

[27] Bacchi E Negri C Zanolin ME Milanese C Faccioli N Trombetta M . Metabolic effects of aerobic training and resistance training in type 2 diabetic subjects: a randomized controlled trial (the RAED2 study). Diabetes Care (2012) 35(4):676–82. doi: 10.2337/dc11-1655 PMC3308269 22344613

[28] Burr JF Rowan CP Jamnik VK Riddell MC . The role of physical activity in type 2 diabetes prevention: physiological and practical perspectives. Phys Sportsmed (2010) 38(1):72–82. doi: 10.3810/psm.2010.04.1764 20424404

[29] Jiang Y Tan S Wang Z Guo Z Li Q Wang J . Aerobic exercise training at maximal fat oxidation intensity improves body composition, glycemic control, and physical capacity in older people with type 2 diabetes. J Exerc Sci Fit (2020) 18(1):7–13. doi: 10.1016/j.jesf.2019.08.003 31641362 PMC6796612

[30] Mann S Beedie C Balducci S Zanuso S Allgrove J Bertiato F . Changes in insulin sensitivity in response to different modalities of exercise: a review of the evidence. Diabetes Metab Res Rev (2014) 30(4):257–68. doi: 10.1002/dmrr.2488 24130081

[31] Strasser B Pesta D . Resistance training for diabetes prevention and therapy: experimental findings and molecular mechanisms. BioMed Res Int (2013) 2013:805217. doi: 10.1155/2013/805217 24455726 PMC3881442

[32] WHO . WHO Guidelines on Physical Activity and Sedentary Behaviour. Geneva: World Health Organization (2020).

[33] Amaro-Gahete FJ De-la OA Jurado-Fasoli L Dote-Montero M Gutierrez A Ruiz JR . Changes in physical fitness after 12 weeks of structured concurrent exercise training, high intensity interval training, or whole-body electromyostimulation training in sedentary middle-aged adults: A randomized controlled trial. Front Physiol (2019) 10:451. doi: 10.3389/fphys.2019.00451 31105580 PMC6492765

[34] Badaam KM Zingade US . The effect of traditional aerobic exercise and sprint interval training on insulin resistance in men with prediabetes: A randomised controlled trial. Cureus (2021) 13(12):e20789. doi: 10.7759/cureus.20789 35141057 PMC8802663

[35] Hurst C Weston KL Weston M . The effect of 12 weeks of combined upper- and lower-body high-intensity interval training on muscular and cardiorespiratory fitness in older adults. Aging Clin Exp Res (2019) 31(5):661–71. doi: 10.1007/s40520-018-1015-9 PMC6491660 30051418

[36] Faerch K Blond MB Bruhn L Amadid H Vistisen D Clemmensen KKB . The effects of dapagliflozin, metformin or exercise on glycaemic variability in overweight or obese individuals with prediabetes (the PRE-D Trial): a multi-arm, randomised, controlled trial. Diabetologia (2021) 64(1):42–55. doi: 10.1007/s00125-020-05306-1 33064182

[37] Liubaoerjijin Y Terada T Fletcher K Boule NG . Effect of aerobic exercise intensity on glycemic control in type 2 diabetes: a meta-analysis of head-to-head randomized trials. Acta Diabetol (2016) 53(5):769–81. doi: 10.1007/s00592-016-0870-0 27255501

[38] Moher D Shamseer L Clarke M Ghersi D Liberati A Petticrew M . Preferred reporting items for systematic review and meta-analysis protocols (PRISMA-P) 2015 statement. Syst Rev (2015) 4:1. doi: 10.1186/2046-4053-4-1 25554246 PMC4320440

[39] Page MJ McKenzie JE Bossuyt PM Boutron I Hoffmann TC Mulrow CD . The PRISMA 2020 statement: an updated guideline for reporting systematic reviews. BMJ (2021) 372:n71. doi: 10.1136/bmj.n71 33782057 PMC8005924

[40] Pilch W Tota L Sadowska-Krepa E Piotrowska A Kepinska M Palka T . The effect of a 12-week health training program on selected anthropometric and biochemical variables in middle-aged women. BioMed Res Int (2017) 2017:9569513. doi: 10.1155/2017/9569513 29130051 PMC5654297

[41] Liao HC Zhong SG Li P Chen WB Cheng C Wang YG . Effects and mechanism of moderate aerobic exercise on impaired fasting glucose improvement. Lipids Health Dis (2015) 14:157. doi: 10.1186/s12944-015-0117-z 26630989 PMC4668668

[42] Viskochil R Malin SK Blankenship JM Braun B . Exercise training and metformin, but not exercise training alone, decreases insulin production and increases insulin clearance in adults with prediabetes. J Appl Physiol (1985) (2017) 123(1):243–8. doi: 10.1152/japplphysiol.00790.2016 PMC5538813 28473613

[43] Faerch K Vistisen D Johansen NB Jorgensen ME . Cardiovascular risk stratification and management in pre-diabetes. Curr Diabetes Rep (2014) 14(6):493. doi: 10.1007/s11892-014-0493-1 24743942

[44] Higgins JPT Green S eds. Cochrane Handbook for Systematic Reviews of Interventions. The Cochrane Collaboration (2011). Available from www.handbook.cochrane.org.

[45] Higgins JPT Thomas J Chandler J Cumpston M Li T Page MJ . Cochrane Handbook for Systematic Reviews of Interventions version 6.0. Chichester (UK): Cochrane (2019). updated July 2019.

[46] Egger M Davey Smith G Schneider M Minder C . Bias in meta-analysis detected by a simple, graphical test. BMJ (1997) 315(7109):629–34. doi: 10.1136/bmj.315.7109.629 PMC2127453 9310563

[47] Huedo-Medina TB Sanchez-Meca J Marin-Martinez F Botella J . Assessing heterogeneity in meta-analysis: Q statistic or I2 index? Psychol Methods (2006) 11(2):193–206. doi: 10.1037/1082-989X.11.2.193 16784338

[48] Owen RK Bradbury N Xin Y Cooper N Sutton A . MetaInsight: An interactive web-based tool for analyzing, interrogating, and visualizing network meta-analyses using R-shiny and netmeta. Res Synth Methods (2019) 10(4):569–81. doi: 10.1002/jrsm.1373 PMC6973101 31349391

[49] Alvarez C Ramirez R Flores M Zuniga C Celis-Morales CA . [Effect of sprint interval training and resistance exercise on metabolic markers in overweight women]. Rev Med Chil (2012) 140(10):1289–96. doi: 10.4067/S0034-98872012001000008 23559286

[50] Burtscher M Gatterer H Kunczicky H Brandstatter E Ulmer H . Supervised exercise in patients with impaired fasting glucose: impact on exercise capacity. Clin J Sport Med (2009) 19(5):394–8. doi: 10.1097/JSM.0b013e3181b8b6dc 19741312

[51] Chen X Zhao S Hsue C Dai X Liu L Miller JD . Effects of aerobic training and resistance training in reducing cardiovascular disease risk for patients with prediabetes: A multi-center randomized controlled trial. Prim Care Diabetes (2021) 15(6):1063–70. doi: 10.1016/j.pcd.2021.08.013 34649825

[52] Desch S Sonnabend M Niebauer J Sixt S Sareban M Eitel I . Effects of physical exercise versus rosiglitazone on endothelial function in coronary artery disease patients with prediabetes. Diabetes Obes Metab (2010) 12(9):825–8. doi: 10.1111/j.1463-1326.2010.01234.x 20649635

[53] Fritz T Caidahl K Krook A Lundstrom P Mashili F Osler M . Effects of Nordic walking on cardiovascular risk factors in overweight individuals with type 2 diabetes, impaired or normal glucose tolerance. Diabetes Metab Res Rev (2013) 29(1):25–32. doi: 10.1002/dmrr.2321 22887834

[54] Gilbertson NM Mandelson JA Hilovsky K Akers JD Hargens TA Wenos DL . Combining supervised run interval training or moderate-intensity continuous training with the diabetes prevention program on clinical outcomes. Eur J Appl Physiol (2019) 119(7):1503–12. doi: 10.1007/s00421-019-04137-2 30980133

[55] Hansen E Landstad BJ Gundersen KT Torjesen PA Svebak S . Insulin sensitivity after maximal and endurance resistance training. J Strength Cond Res (2012) 26(2):327–34. doi: 10.1519/JSC.0b013e318220e70f 22240549

[56] Herzig KH Ahola R Leppaluoto J Jokelainen J Jamsa T Keinanen-Kiukaanniemi S . Light physical activity determined by a motion sensor decreases insulin resistance, improves lipid homeostasis and reduces visceral fat in high-risk subjects: PreDiabEx study RCT. Int J Obes (Lond) (2014) 38(8):1089–96. doi: 10.1038/ijo.2013.224 PMC4125749 24285336

[57] Liu Y Li J Zhang Z Tang Y Chen Z Wang Z . Effects of exercise intervention on vascular endothelium functions of patients with impaired glucose tolerance during prediabetes mellitus. Exp Ther Med (2013) 5(6):1559–65. doi: 10.3892/etm.2013.1064 PMC3702621 23837031

[58] Malin SK Gerber R Chipkin SR Braun B . Independent and combined effects of exercise training and metformin on insulin sensitivity in individuals with prediabetes. Diabetes Care (2012) 35(1):131–6. doi: 10.2337/dc11-0925 PMC3241331 22040838

[59] Rowan CP Riddell MC Gledhill N Jamnik VK . Aerobic exercise training modalities and prediabetes risk reduction. Med Sci Sports Exerc (2017) 49(3):403–12. doi: 10.1249/MSS.0000000000001135 27776003

[60] Slentz CA Bateman LA Willis LH Granville EO Piner LW Samsa GP . Effects of exercise training alone vs a combined exercise and nutritional lifestyle intervention on glucose homeostasis in prediabetic individuals: a randomised controlled trial. Diabetologia (2016) 59(10):2088–98. doi: 10.1007/s00125-016-4051-z PMC5026926 27421729

[61] Venojarvi M Wasenius N Manderoos S Heinonen OJ Hernelahti M Lindholm H . Nordic walking decreased circulating chemerin and leptin concentrations in middle-aged men with impaired glucose regulation. Ann Med (2013) 45(2):162–70. doi: 10.3109/07853890.2012.727020 23110613

[62] Yuan X Dai X Liu L Hsue C Miller JD Fang Z . Comparing the effects of 6 months aerobic exercise and resistance training on metabolic control and beta-cell function in Chinese patients with prediabetes: A multicenter randomized controlled trial. J Diabetes (2020) 12(1):25–37. doi: 10.1111/1753-0407.12955 31141300

[63] Burtscher M Gatterer H Dünnwald T Pesta D Faulhaber M Netzer N . Effects of supervised exercise on gamma-glutamyl transferase levels in patients with isolated impaired fasting glucose and those with impaired fasting glucose plus impaired glucose tolerance. Exp Clin Endocrinol Diabetes (2012) 120(8):445–50. doi: 10.1055/s-0032-1311642 22639399

[64] Colberg SR Sigal RJ Fernhall B Regensteiner JG Blissmer BJ Rubin RR . Exercise and type 2 diabetes: the American College of Sports Medicine and the American Diabetes Association: joint position statement. Diabetes Care (2010) 33(12):e147–167. doi: 10.2337/dc10-9990 PMC2992225 21115758

[65] Heiskanen MA Sjoros TJ Heinonen IHA Loyttyniemi E Koivumaki M Motiani KK . Sprint interval training decreases left-ventricular glucose uptake compared to moderate-intensity continuous training in subjects with type 2 diabetes or prediabetes. Sci Rep (2017) 7(1):10531. doi: 10.1038/s41598-017-10931-9 28874821 PMC5585392

[66] Jung ME Bourne JE Beauchamp MR Robinson E Little JP . High-intensity interval training as an efficacious alternative to moderate-intensity continuous training for adults with prediabetes. J Diabetes Res (2015) 2015:191595. doi: 10.1155/2015/191595 25918728 PMC4396724

[67] Little JP Jung ME Wright AE Wright W Manders RJ . Effects of high-intensity interval exercise versus continuous moderate-intensity exercise on postprandial glycemic control assessed by continuous glucose monitoring in obese adults. Appl Physiol Nutr Metab (2014) 39(7):835–41. doi: 10.1139/apnm-2013-0512 24773254

[68] Borg GA . Psychophysical bases of perceived exertion. Med Sci Sports Exerc (1982) 14(5):377–81. doi: 10.1249/00005768-198205000-00012 7154893

[69] Williams N . The borg rating of perceived exertion (RPE) scale. Occup Med (2017) 67(5):404–5. doi: 10.1093/occmed/kqx063

[70] De Nardi AT Tolves T Lenzi TL Signori LU Silva A . High-intensity interval training versus continuous training on physiological and metabolic variables in prediabetes and type 2 diabetes: A meta-analysis. Diabetes Res Clin Pract (2018) 137:149–59. doi: 10.1016/j.diabres.2017.12.017 29329778

[71] Robinson E Durrer C Simtchouk S Jung ME Bourne JE Voth E . Short-term high-intensity interval and moderate-intensity continuous training reduce leukocyte TLR4 in inactive adults at elevated risk of type 2 diabetes. J Appl Physiol (1985) (2015) 119(5):508–16. doi: 10.1152/japplphysiol.00334.2015 PMC4556835 26139217

[72] Huang L Fang Y Tang L . Comparisons of different exercise interventions on glycemic control and insulin resistance in prediabetes: a network meta-analysis. BMC Endocr Disord (2021) 21(1):181. doi: 10.1186/s12902-021-00846-y 34488728 PMC8422751

[73] Liu L Ma X Xu H Ruan S Yuan X . Comparing the effects of 12 months aerobic exercise and resistance training on glucose metabolism among prediabetes phenotype: A explorative randomized controlled trial. Prim Care Diabetes (2021) 15(2):340–6. doi: 10.1016/j.pcd.2020.11.003 33309489

[74] Yang Z Scott CA Mao C Tang J Farmer AJ . Resistance exercise versus aerobic exercise for type 2 diabetes: a systematic review and meta-analysis. Sports Med (2014) 44(4):487–99. doi: 10.1007/s40279-013-0128-8 24297743

[75] Bweir S Al-Jarrah M Almalty AM Maayah M Smirnova IV Novikova L . Resistance exercise training lowers HbA1c more than aerobic training in adults with type 2 diabetes. Diabetol Metab Syndr (2009) 1:27. doi: 10.1186/1758-5996-1-27 20003276 PMC2800839

[76] Lee J Kim D Kim C . Resistance training for glycemic control, muscular strength, and lean body mass in old type 2 diabetic patients: A meta-analysis. Diabetes Ther (2017) 8(3):459–73. doi: 10.1007/s13300-017-0258-3 PMC5446383 28382531

[77] Gordon BA Benson AC Bird SR Fraser SF . Resistance training improves metabolic health in type 2 diabetes: a systematic review. Diabetes Res Clin Pract (2009) 83(2):157–75. doi: 10.1016/j.diabres.2008.11.024 19135754

[78] Irvine C Taylor NF . Progressive resistance exercise improves glycaemic control in people with type 2 diabetes mellitus: a systematic review. Aust J Physiother (2009) 55(4):237–46. doi: 10.1016/S0004-9514(09)70003-0 19929766

[79] Sigal RJ Kenny GP Boule NG Wells GA Prud'homme D Fortier M . Effects of aerobic training, resistance training, or both on glycemic control in type 2 diabetes: a randomized trial. Ann Intern Med (2007) 147(6):357–69. doi: 10.7326/0003-4819-147-6-200709180-00005 17876019

[80] Winding KM Munch GW Iepsen UW Van Hall G Pedersen BK Mortensen SP . The effect on glycaemic control of low-volume high-intensity interval training versus endurance training in individuals with type 2 diabetes. Diabetes Obes Metab (2018) 20(5):1131–9. doi: 10.1111/dom.13198 29272072

[81] Galmes-Panades AM Bennasar-Veny M Oliver P Garcia-Coll N Chaplin A Fresneda S . Efficacy of different modalities and frequencies of physical exercise on glucose control in people with prediabetes (GLYCEX randomised trial). Metabolites (2022) 12(12):1286. doi: 10.3390/metabo12121286 36557324 PMC9785307

[82] American Diabetes Association . Diagnosis and classification of diabetes mellitus. Diabetes Care (2012) 35 Suppl 1:S64–71. doi: 10.2337/dc12-s064 PMC3632174 22187472

[83] NICE . Preventing type 2 diabetes: risk identification and interventions for individuals at high risk. England: NICE public health guidance (2012).

[84] Diabetes Canada Clinical Practice Guidelines Expert Committee Punthakee Z Goldenberg R Katz P . Definition, classification and diagnosis of diabetes, prediabetes and metabolic syndrome. Can J Diabetes (2018) 42 Suppl 1:S10–5. doi: 10.1016/j.jcjd.2017.10.003 29650080

[85] Kim CH Kim HK Kim EH Bae SJ Choe J Park JY . Risk of progression to diabetes from prediabetes defined by HbA1c or fasting plasma glucose criteria in Koreans. Diabetes Res Clin Pract (2016) 118:105–11. doi: 10.1016/j.diabres.2016.06.009 27368062

[86] World Health Organization (WHO) . Use of glycated haemoglobin (HbA1c) in the diagnosis of diabetes mellitus. In: Pess W , editor. Abbreviated Report of a WHO Consultation. Geneva: WHO. (2011). 26158184

