# Supplementary material for: Effect of physical activity and different exercise modalities on glycemic control in people with prediabetes: a systematic review and meta-analysis of randomized controlled trials
Source: Front Endocrinol (Lausanne). 2023 Sep 28;14:1233312. doi: 10.3389/fendo.2023.1233312 (PMC10569497; doi:10.3389/fendo.2023.1233312)
Supplement: Supplementary file 1 [file DataSheet_1.pdf]

Supplementary information

Additional file 1. Exemplary literature search

MEDLINE/PubMed: Search on June 23, 2022; 485 references

#1 (("prediabet*") AND (("exercise") OR "physical activity")) AND (“trial”))

#2 (("Prediabetic state"[Mesh]) OR "Prediabetes" OR “pre-diabetes” OR “intermediate hyperglycemia” OR "glucose intolerance" OR "impaired glucose tolerance" OR "impaired fasting glucose") AND (("Exercise Therapy"[Mesh]) OR "muscle strength" OR "muscle strengthening" OR "weight lifting" OR "weight-lifting" OR "weight bearing" OR "weight-bearing" OR "weight training" OR "circuit training" OR "strength exercise" OR "strengthening exercise" OR "strength training" OR "resistance exercise" OR "resistance training" OR "progressive resistance" OR "Physical Exercise" OR "Isometric Exercise" OR "aerobic exercise" OR "aerobic training" OR "exercise therapy") AND trial*

#3 ("Prediabetic state"[Mesh] OR "Prediabetes"[All Fields] OR "pre-diabetes"[All Fields] OR " intermediate hyperglycemia"[All Fields] OR "glucose intolerance"[All Fields] OR " impaired glucose tolerance"[All Fields]) AND ("Exercise Therapy"[Mesh] OR " muscle strength"[All Fields] OR "muscle strengthening"[All Fields] OR "weight lifting"[All Fields] OR "weight-lifting"[All Fields] OR "weight bearing"[All Fields] OR "weight-bearing"[All Fields] OR "weight training"[All Fields] OR "circuit training"[All Fields] OR "strength exercise"[All Fields] OR "strengthening exercise"[All Fields] OR "strength training"[All Fields] OR "resistance exercise"[All Fields] OR "resistance training"[All Fields] OR "progressive resistance"[All Fields] OR "Physical Exercise"[All Fields] OR "Isometric Exercise"[All Fields] OR "aerobic exercise"[All Fields] OR "aerobic training"[All Fields] OR "exercise therapy"[All Fields] OR "interval training"[All Fields] OR "training"[All Fields] OR "resistance training"[All Fields]) AND ("Randomized Controlled Trials"[Mesh] OR "trial" [All Fields])

EMBASE (via Elsevier): Search on June 23, 2022; 793 references

#1 ('prediabetic state'/exp OR 'prediabetes' OR 'pre-diabetes' OR 'intermediate hyperglycemia' OR 'glucose* intolerance' OR 'impaired* glucose* tolerance' OR 'impaired* fasting* glucose')

#2 ('Exercise Therapy'/exp OR ' muscle strength' OR 'muscle strengthening' OR 'weight lifting' OR 'weight-lifting' OR 'weight bearing' OR weight-bearing OR 'weight training' OR 'circuit training' OR 'strength exercise' OR 'strengthening exercise' OR 'strength training' OR 'resistance exercise' OR 'resistance training' OR 'progressive resistance' OR 'Physical Exercise' OR 'Isometric Exercise' OR 'aerobic exercise' OR 'aerobic training' OR 'exercise therapy' OR 'interval training' OR training OR 'resistance training')

#3 #1 AND #2

#4 ('Prediabetic state'/exp OR Prediabetes OR pre-diabetes OR ' intermediate hyperglycemia' OR 'glucose intolerance' OR ' impaired glucose tolerance') AND ('Exercise Therapy'/exp OR ' muscle strength' OR 'muscle strengthening' OR 'weight lifting' OR 'weight-lifting' OR 'weight bearing' OR weight-bearing OR 'weight training' OR 'circuit training' OR 'strength exercise' OR 'strengthening exercise' OR 'strength training' OR 'resistance exercise' OR 'resistance training' OR 'progressive

resistance' OR 'Physical Exercise' OR 'Isometric Exercise' OR 'aerobic exercise' OR 'aerobic training' OR 'exercise therapy' OR 'interval training' OR training OR 'resistance training') AND ('Randomized Controlled Trials'/exp OR trial)

Web of Science: Search on June 23, 2022; 447 references

("Prediabetic state" OR Prediabetes OR pre-diabetes OR " intermediate hyperglycemia" OR "glucose intolerance" OR " impaired glucose tolerance") AND ("Exercise Therapy" OR " muscle strength" OR "muscle strengthening" OR "weight lifting" OR weight-lifting OR "weight bearing" OR weight-bearing OR "weight training" OR "circuit training" OR "strength exercise" OR "strengthening exercise" OR "strength training" OR "resistance exercise" OR "resistance training" OR "progressive resistance" OR "Physical Exercise" OR "Isometric Exercise" OR "aerobic exercise" OR "aerobic training" OR "exercise therapy" OR "interval training" OR training OR "resistance training") AND ("Randomized Controlled Trials" OR trial)

SPORTDiscus (via the EBSCO): Search on June 23, 2022; 974 references

(DE "Prediabetic state" OR TX "Prediabetes" OR TX "pre-diabetes" OR TX " intermediate hyperglycemia" OR TX "glucose intolerance" OR TX " impaired glucose tolerance") AND (DE "Exercise Therapy" OR TX " muscle strength" OR TX "muscle strengthening" OR TX "weight lifting" OR TX "weight-lifting" OR TX "weight bearing" OR TX "weight-bearing" OR TX "weight training" OR TX "circuit training" OR TX "strength exercise" OR TX "strengthening exercise" OR TX "strength training" OR TX "resistance exercise" OR TX "resistance training" OR TX "progressive resistance" OR TX "Physical Exercise" OR TX "Isometric Exercise" OR TX "aerobic exercise" OR TX "aerobic training" OR TX "exercise therapy" OR TX "interval training" OR TX "training" OR TX "resistance training") AND (DE "Randomized Controlled Trials" OR TX "trial")

Cochrane databases: Search on June 23, 2022; 462 references

#1 “Prediabetic state" OR "Prediabetes" OR “pre-diabetes” OR “intermediate hyperglycemia” OR "glucose* intolerance" OR "impaired* glucose* tolerance" OR "impaired* fasting* glucose”: ti,ab,kw (Word variations have been searched)

#2 MeSH descriptor: [Prediabetes state] explode all trees

#3 #1 OR #2

#4 "Physical exercise" OR "exercise therapy" OR "muscle strength*" OR "weight lifting" OR "weight-lifting" OR "weight bearing" OR "weight-bearing" OR "weight training" OR "circuit training" OR "strength exercise" OR "strengthening exercise" OR "strength training" OR "resistance exercise" OR "resistance training" OR "progressive resistance" OR "Isometric Exercise" OR "aerobic exercise" OR "aerobic training" OR “Physical activity”: ti,ab,kw (Word variations have been searched)

#5 MeSH descriptor: [Exercise Therapy] explode all trees

#6 #4 OR #5

#7 #3 AND #6

#8 ([mh "Prediabetic state"] OR Prediabetes OR pre-diabetes OR " intermediate hyperglycemia" OR "glucose intolerance" OR " impaired glucose tolerance") AND ([mh "Exercise Therapy"] OR " muscle strength" OR "muscle strengthening" OR "weight lifting" OR weight-lifting OR "weight bearing" OR weight-bearing OR "weight training" OR "circuit training" OR "strength exercise" OR "strengthening exercise" OR "strength training" OR "resistance exercise" OR "resistance training" OR "progressive resistance" OR "Physical Exercise" OR "Isometric Exercise" OR "aerobic exercise" OR "aerobic training" OR "exercise therapy" OR "interval training" OR training OR "resistance training") AND ([mh "Randomized Controlled Trials"] OR trial)

Additional file 2. Glossary of terms

Physical activity is defined as any bodily movement produced by skeletal muscles contraction, that increase the energy expenditure [1-4]. The terms physical activity, exercise and sports are often used interchangeably, but these are not exactly the same concepts. The concept of physical activity is more general and encompasses the concepts of exercise and sport.

Physical activity is composed of four **dimensions**, these are: modality, frequency, duration and intensity. **Modality** is the specific activity performed (i.e., aerobic activities, anaerobic activities, strength training or balance). **Frequency** is the number of sessions per day or per week that are made. **Duration** is the time in hours or minutes of the activity during a specified time frame such a day or a week. Most studies suggest that the duration must be at least 10 minutes to get any beneficial effects on health [5]. **Intensity** is an indicator of the metabolic demand of an activity. The higher the intensity, the more effort is required to achieve the activity. Intensity can be objectively quantified with physiological measures (i.e., heart rate or oxygen consumption among others), subjectively assessed by perceptual characteristics (i.e., talk test or scale of perceived exertion), or quantified by body movement (i.e., 3-axial body accelerations) [2].
Physical activity intensity can be classified as light, moderate and vigorous according to the magnitude of the effort required to overcome the activity. **Light physical activity** lets the person talk or sing without effort during the practice, such as a slow walk. **Moderate physical activity** (MPA) is an activity that requires a moderate amount of effort, increases sweating and noticeably accelerates heart and breathing rate [6]. **Vigorous physical activity** (VPA) is an activity that requires a large amount of effort, increases sweating and causes rapid breathing and substantial increase in heart rate [6]. **Moderate-to-vigorous physical activity** (MVPA) are activities that combine both intensities, usually depending on the speed at which the activity is performed, e.g. swimming or cycling. They can be differentiated by the 'talk test': being able to talk but not sing indicates MPA, while having difficulty talking without pausing is a sign of VPA [6].

Physical inactivity is an insufficient physical activity level to meet physical activity recommendations [4].

Exercise is a type of physical activity, characterized for being structured, planned, and practiced with repetitive bodily movements, aimed to perform or maintain physical fitness (a set of personal characteristics to perform physical activity) [4, 7].

Sports are all forms of competitive physical activity or games, individual or played in team, that can be casual or organized, and are aimed to maintain or improve physical ability and skills while providing enjoyment (i.e. basketball, tennis or canoeing) [5].

Aerobic training (AT) is characterised by training at an intensity that allows the aerobic route to be used as a metabolic route for obtaining energy, in which the body's large muscles move in a rhythmic manner for a sustained period of time. Aerobic Training can also called aerobic activity or endurance activity, and improves cardiorespiratory fitness.

Some examples for AT are: running, brisk walking, swimming or cycling [4, 8]. With AT there is an improvement in insulin sensitivity [9].

Anaerobic physical activity consists of brief intense bursts of exercise, such as weightlifting and sprints, where oxygen demand surpasses oxygen supply, and consequently, the lactic anaerobic or a lactic anaerobic metabolic pathway is used to obtain energy [4].

Interval training (IT) is a form of physical training that consists of short periods of exercise of varying intensity with short periods of rest in between. One study found that this type of exercise strategy was associated with glycogen depletion, which may induce improvements in insulin sensitivity [11, 12].

High Intensity Interval Training (HIIT) is a type of interval training, characterised by being performed at high intensity. High Intensity Interval Training is a very vigorous physical activity performed in short bursts interspersed with breaks [6]. In the present review, has been considered as a HIIT those interval training interventions that were performed at high intensity, being considered high intensity those with a heart rate ≥85% or a VO_2Max ≥60% [13-17].

Resistance training (RT) is also referred to as muscle strengthening. We defined RT as exercise performed against some type of resistance to increase muscle strength, muscle endurance or muscle power. Resistance training exercises can be performed by weightlifting using either machine weights, free weights or with elastic bands that resist movement. Some examples of RT are: bench press, seated row, shoulder press, leg press, or weight strength [8]. Resistance training regimens should include multi-joint exercises that affect more than one muscle group (i.e., lower back extension, chest press, shoulder press, pull-down, dips, leg press or squats). Perform exercise involving major muscle groups is also important, such as quadriceps extensions, leg curls, biceps curls and triceps extensions [7, 10]. Research findings show that this intervention strategy also increases insulin sensitivity and glucose tolerance primarily through increased skeletal muscle mass [7].

References

1. Thompson PD, Arena R, Riebe D, Pescatello LS, American College of Sports M: **ACSM's new preparticipation health screening recommendations from ACSM's guidelines for exercise testing and prescription, ninth edition**. *Curr Sports Med Rep* 2013, **12**(4):215-217.
2. Strath SJ, Kaminsky LA, Ainsworth BE, Ekelund U, Freedson PS, Gary RA, Richardson CR, Smith DT, Swartz AM, American Heart Association Physical Activity Committee of the Council on L *et al*: **Guide to the assessment of physical activity: Clinical and research applications: a scientific statement from the American Heart Association**. *Circulation* 2013, **128**(20):2259-2279.
3. Caspersen CJ, Powell KE, Christenson GM: **Physical activity, exercise, and physical fitness: definitions and distinctions for health-related research**. *Public Health Rep* 1985, **100**(2):126-131.
4. WHO. In: *WHO Guidelines on Physical Activity and Sedentary Behaviour.* edn. Geneva; 2020.
5. WHO. In: *Pacific Physical Activity Guidelines for Adults: Framework for Accelerating the Communication of Physical Activity Guidelines.* edn. Geneva; 2008.
6. Davies DSC, Atherton F, McBride M, Calderwood C: **UK Chief Medical Officers' Physical Activity Guidelines**. In.; 2009.

7. American College of Sports M: **American College of Sports Medicine position stand. Progression models in resistance training for healthy adults**. *Med Sci Sports Exerc* 2009, **41**(3):687-708.
8. Pan B, Ge L, Xun YQ, Chen YJ, Gao CY, Han X, Zuo LQ, Shan HQ, Yang KH, Ding GW *et al*: **Exercise training modalities in patients with type 2 diabetes mellitus: a systematic review and network meta-analysis**. *Int J Behav Nutr Phys Act* 2018, **15**(1):72.
9. Roberts CK, Hevener AL, Barnard RJ: **Metabolic syndrome and insulin resistance: underlying causes and modification by exercise training**. *Compr Physiol* 2013, **3**(1):1-58.
10. Garber CE, Blissmer B, Deschenes MR, Franklin BA, Lamonte MJ, Lee IM, Nieman DC, Swain DP, American College of Sports M: **American College of Sports Medicine position stand. Quantity and quality of exercise for developing and maintaining cardiorespiratory, musculoskeletal, and neuromotor fitness in apparently healthy adults: guidance for prescribing exercise**. *Med Sci Sports Exerc* 2011, **43**(7):1334-1359.
11. Korhonen M, Halmesmaki K, Lepantalo M, Venermo M: **Predictors of failure of endovascular revascularization for critical limb ischemia**. *Scand J Surg* 2012, **101**(3):170-176.
12. Whyte LJ, Gill JM, Cathcart AJ: **Effect of 2 weeks of sprint interval training on health-related outcomes in sedentary overweight/obese men**. *Metabolism* 2010, **59**(10):1421-1428.
13. Colberg SR, Sigal RJ, Fernhall B, Regensteiner JG, Blissmer BJ, Rubin RR, Chasan-Taber L, Albright AL, Braun B, American College of Sports M *et al*: **Exercise and type 2 diabetes: the American College of Sports Medicine and the American Diabetes Association: joint position statement**. *Diabetes Care* 2010, **33**(12):e147-167.
14. Heiskanen MA, Sjoros TJ, Heinonen IHA, Loyttyniemi E, Koivumaki M, Motiani KK, Eskelinen JJ, Virtanen KA, Knuuti J, Hannukainen JC *et al*: **Sprint interval training decreases left-ventricular glucose uptake compared to moderate-intensity continuous training in subjects with type 2 diabetes or prediabetes**. *Sci Rep* 2017, **7**(1):10531.
15. Jung ME, Bourne JE, Beauchamp MR, Robinson E, Little JP: **High-intensity interval training as an efficacious alternative to moderate-intensity continuous training for adults with prediabetes**. *J Diabetes Res* 2015, **2015**:191595.
16. Little JP, Gillen JB, Percival ME, Safdar A, Tarnopolsky MA, Punthakee Z, Jung ME, Gibala MJ: **Low-volume high-intensity interval training reduces hyperglycemia and increases muscle mitochondrial capacity in patients with type 2 diabetes**. *J Appl Physiol (1985)* 2011, **111**(6):1554-1560.
17. Little JP, Jung ME, Wright AE, Wright W, Manders RJ: **Effects of high-intensity interval exercise versus continuous moderate-intensity exercise on postprandial glycemic control assessed by continuous glucose monitoring in obese adults**. *Appl Physiol Nutr Metab* 2014, **39**(7):835-841.
